# Overcoming the clinical challenges of traditional ayahuasca: a first-in-human trial exploring novel routes of administration of N,N-Dimethyltryptamine and harmine 

**DOI:** 10.3389/fphar.2023.1246892

**Published:** 2023-11-27

**Authors:** Dario A. Dornbierer, Laurenz Marten, Jovin Mueller, Helena D. Aicher, Michael J. Mueller, Martina Boxler, Michael Kometer, Davor Kosanic, Robin von Rotz, Maxim Puchkov, Thomas Kraemer, Hans-Peter Landolt, Erich Seifritz, Milan Scheidegger

**Affiliations:** ^1^ Institute of Pharmacology and Toxicology, University of Zurich, Zurich, Switzerland; ^2^ Department of Forensic Pharmacology and Toxicology, Zurich Institute of Forensic Medicine, University of Zurich, Zurich, Switzerland; ^3^ Department of Psychiatry, Psychotherapy and Psychosomatics, University Hospital of Psychiatry Zurich, Zurich, Switzerland; ^4^ Department of Chemistry and Applied Biosciences, Zurich, Switzerland; ^5^ Department of Psychology, University of Zurich, Zurich, Switzerland; ^6^ Neuroscience Center Zurich, University of Zurich and ETH Zurich, Zurich, Switzerland; ^7^ Department of Health Science & Technology, Zurich, Switzerland; ^8^ Reconnect Labs, Winterthur, Switzerland; ^9^ Institute of Pharmaceutical Technology, University of Basel, Basel, Switzerland

**Keywords:** DMT, harmine, ayahuasca, psychedelic, formulations

## Abstract

Recently, the Amazonian plant medicine “ayahuasca”—containing the psychedelic compound N,N-dimethyltryptamine (DMT) and numerous β-carboline alkaloids, such as harmine—has been suggested to exhibit beneficial effects in patients with affective and other mental health disorders. Although ayahuasca ingestion is considered safe, its pharmacokinetics/pharmacodynamics and tolerability profile pose some challenges and may limit the clinical applicability in vulnerable patient populations. While overdosing and the admixture of intolerable plant constituents may explain some of the common adverse reactions, the peroral route of administration may represent another relevant source of gastro-intestinal intolerabilities and unpredictable pharmacokinetics across users. To overcome these challenges, the present work aimed at creating ayahuasca-analogue formulations with improved pharmacokinetics and tolerability profiles. To this end, we developed peroral formulas and compared them with parenteral formulas specifically designed to circumvent the gastro-intestinal tract. In more detail, peroral administration of a capsule (containing purified DMT and harmine) was tested against a combined administration of an oromucosal harmine tablet and an intranasal DMT spray at two dose levels in an open-label within-subject study in 10 healthy male subjects. Pharmacokinetic and pharmacodynamic profiles were assessed by means of continuous blood sampling, vital sign monitoring, and psychometric assessments. Common side effects induced by traditional herbal ayahuasca such as nausea, vomiting, and diarrhea were significantly attenuated by our DMT/harmine formulations. While all preparations were well tolerated, the combined buccal/intranasal administration of harmine and DMT yielded substantially improved pharmacokinetic profiles, indicated by significantly reduced variations in systemic exposure. In conclusion, the combined buccal/intranasal administration of harmine and DMT is an innovative approach that may pave the way towards a safe, rapid-acting, and patient-oriented administration of DMT/harmine for the treatment of affective disorders.

**Clinical Trial Registration:** clinicaltrials.gov, identifier NCT04716335

## 1 Introduction

Affective disorders are widespread in society and leading causes of disability and ill health worldwide. Despite the high prevalence, most of the available antidepressant therapies show suboptimal efficacy (Kirsch et al., 2008; Munkholm et al., 2019) and are currently prescribed in a lengthy trial and error approach that requires weeks or months to see the clinical benefit. Still, fewer than 50% of all patients with depression show full remission with optimized standard treatment, and often, these medications have considerable side effects (Trivedi et al., 2008). Thus, an urgent and unmet need exists for more rapid-acting, efficacious, and sustainable therapies.

Promising novel directions include the rediscovery of psychedelic substances for the treatment of various mental health disorders (Nichols et al., 2017). The glutamatergic NMDA receptor antagonist and dissociative anesthetic esketamine was recently FDA-approved as a prescription nasal spray for treatment-resistant depression (Zarate et al., 2006; Naughton et al., 2014). Notably, classical serotonergic psychedelics such as LSD (Holze et al., 2023) and psilocybin (Goodwin et al., 2022; von Rotz et al., 2023) were shown to alleviate symptoms of anxiety and depression in a rapid-acting manner with sustained improvements even weeks after a single administration. While the mode of action underlying the rapid-acting effects of psychedelics is widely unknown, their ability to transiently disrupt maladaptive brain dynamics may represent one promising therapeutic factor (Carhart-Harris and Friston, 2019), heralding a paradigmatic shift from substitution-based pharmacotherapy towards transformation-oriented approaches (Scheidegger, 2021). While the clinical potential of psychedelic compounds for the treatment of mental illnesses is being increasingly recognized, their clinical translation potential is compromised by major pharmacological shortcomings. The long duration of action of LSD (10–12 h) and psilocybin (4–6 h) involves the risk of exhausting vulnerable patients and is associated with lengthy and expensive medical surveillance. Moreover, both LSD and psilocybin show rapid induction of tolerance, making them less suited for repeated dosing regimens (Passie et al., 2002; Passie et al., 2008). By contrast, repeated administration of ketamine was shown to sustain antidepressant effects but puts patients at risk due to its addictive potential (Liu et al., 2016).

Recently, the Amazonian plant medicine “ayahuasca” - containing a mixture of N,N-dimethyltryptamine (DMT) and numerous β-carboline alkaloids - has been suggested to exhibit beneficial effects in patients with mental and somatic illnesses (Domínguez-Clavé et al., 2016; Frecska et al., 2016). Ayahuasca has been used for centuries as traditional indigenous medicine in Latin American regions, but its consumption is currently spreading around the world outside of indigenous contexts (Tupper, 2008). Ayahuasca is typically prepared by a decoction of the stems of the Banisteriopsis caapi vine and the leaves of the Psychotria viridis shrub, although hundreds of species are used in addition or as substitutes. Clinical trials show that ayahuasca has equally rapid but more sustainable antidepressant properties compared to ketamine without inducing pharmacological tolerance (dos Santos et al., 2016; Osório et al., 2015; Palhano-Fontes et al., 2019; Sanches et al., 2016). While DMT (e.g., from Psychotria viridis) mediates most of the psychedelic effects of ayahuasca, β-carbolines (e.g., from Banisteriopsis caapi)—such as harmine, harmaline, and tetrahydroharmine—act as competitive MAO-A inhibitors, preventing the metabolic degradation of DMT, thus increasing its bioavailability and prolonging its half live (Callaway et al., 1996). Compared to the rapid onset and experiential intensity of intravenous DMT (Strassman et al., 1994), the subjective effects of orally administered DMT in ayahuasca preparations appear in a slower progressive way and disappear after 3–4 h (dos Santos et al., 2012).

Although ayahuasca ingestion is considered safe (Barbosa et al., 2012), it bears the unpredictable potential to induce several distressing effects, including strong nausea, vomiting, diarrhea, and overwhelming hallucinations. While in the traditional indigenous context of use, the “purgative” effects are subsumed as part of the therapeutic mode of action of ayahuasca (Fotiou and Gearin, 2019), in the Western medical context, they are seen as adverse effects that compromise the clinical pharmacological profile of ayahuasca for regulatory approval as a standardized treatment. We hereby acknowledge the cross-cultural differences between indigenous and biomedical treatment approaches. While we do not aim to neglect the diversity of traditional ayahuasca practices, we are using the term “adverse effects” according to the Western medical understanding of pharmacological side effects, which include nausea and vomiting.

Most of those adverse effects can be attributed to suboptimal pharmacokinetic and pharmacodynamic (PKPD) properties of traditional ayahuasca, overdosing events, and the admixture of intolerable plant materials (with unknown or adverse toxicity). Moreover, the batch-to-batch variability in alkaloid content and considerable interindividual differences in gastrointestinal (GI) absorption and first-pass metabolism complicate the determination of an adequate therapeutic dose, increasing the risk of underdosing, or overdosing with potentially distressing effects. In addition, DMT is readily absorbed into the bloodstream following oral ingestion of ayahuasca, which can cause rapid changes in the consumer’s state of consciousness, triggering distressing and unfavorable psychosomatic reactions.

Based on these considerations, it is proposed that the safety and tolerability profile, and thus, the clinical translation potential of ayahuasca may be improved by the following pharmaceutical adjustments: First, the use of purified forms of DMT and harmine instead of herbal extracts is expected to enable precise dosing, avoiding plant constituents with subordinate therapeutic roles and unfavorable tolerability profiles. Second, by-passing the GI tract is presumed to reduce interindividual differences in bioavailability caused by differences in GI absorption and hepatic first-pass metabolism, increasing the predictability of the drug effects. Moreover, circumventing the GI tract is assumed to spare intestinal 5-HT3 chemoreceptors on the terminals of vagal afferents, thus attenuating nausea, vomiting, and diarrhea. Third, flattening the absorption profile of DMT is expected to moderate the onset of psychotropic effects and thus improve the psychological tolerability of the intervention.

To test our hypotheses, we explored novel administration routes and delivery mechanisms specifically designed to deliver purified forms of DMT and harmine systemically, bypassing the GI tract. The oral administration of DMT with harmaline, harmine, or pharmaceutical MAO inhibitors (e.g., moclobemide) is commonly referred to as “pharmahuasca” or ayahuasca-analogue (Barker, 2022); however, no clinical trials of combined DMT/harmine formulations have been conducted so far in humans to demonstrate its medical use. To avoid the shortcomings of oral dosing due to the first-pass effect, harmine was incorporated into oromucosal tablets for buccal delivery. In contrast, DMT was formulated as an intranasal spray solution to enable metered, incremental dosing. It is hypothesized that this delivery mechanism will significantly advance the precision, predictability, and overall safety and tolerability profiles of DMT/harmine for both basic research and clinical trials.

In this first-in-human clinical trial, peroral administration of DMT and harmine as a capsule was tested against the combined administration of an oromucosal harmine tablet and an intranasal DMT spray at two dose levels in an open-label within-subject pilot study in 10 healthy male subjects. For both approaches, purified DMT hemifumarate (>98.5%, isolated from Mimosa tenuiflora root bark) and synthetic harmine HCl (>98%) were used. PKPD profiles were assessed through continuous blood sampling, vital sign monitoring, and psychometric assessments.

In sum, this study applies pharmaceutical technologies to psychedelic psychopharmacology to advance basic research on the mechanisms of action of ayahuasca and its clinical translation to patient use.

## 2 Methods

### 2.1 Participants and permission

A total of ten healthy male volunteers (30.7 ± 5.4 years) participated in all 4 study days. Following criteria were required for inclusion: male sex to avoid the potential impact of menstrual cycle on blood biochemistry, age within the range of 20–40 years, Body Mass Index between 18.5–25, no current or previous history of somatic, neurological, or psychiatric disorder, no family history of Axis-I psychiatric disorders, no acute or chronic medication intake, no history or current drug abuse (lifetime use >5 occasions, with the exception of occasional cannabis use). The study was approved by the Cantonal Ethics Committee of the Canton of Zurich (Basec-Nr. 2018–01385) and the Swiss Federal Office of Public Health (BAG-Nr. (AB)-8/5-BetmG-2019/009268). All participants provided written informed consent according to the declaration of Helsinki. All participants were financially compensated for the completion of the study.

### 2.2 Study design and setting

**FIGURE 1:**
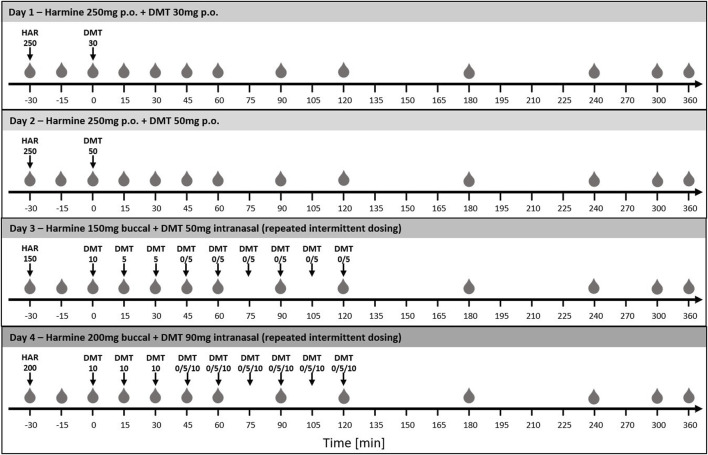
Illustration of the study procedures of days 1–4. Timepoints of blood withdrawal are indicated as grey drops (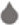). On all study days, harmine was applied 30 min before DMT dosing to provide sufficient MAO-A inhibition at the timepoint of DMT administration. On study days 1 and 2, harmine (HAR) and DMT were administered orally. On days 3 and 4, harmine was given buccally, whereas DMT was administered intranasally in a repeated-intermittent manner. Thereby, volunteers were allowed to self-determine the administered dose based on their general wellbeing, namely, on timepoints 45, 60, 75, 90, 105, and 120 min. On day 3, either 0 or 5 mg, and on day 4, either 0, 5, or 10 mg were allowed. A summary of the chosen dose increments is given in Section 3.1, Table 1.

In this non-random open-label dose-finding within-subject study, we tested four different drug doses and two different administration routes with at least a 1-week washout between intervention days. An overview can be found in Figure 1. The study was conducted during the day in the Human Sleep Laboratories of the University of Zurich. The soundproof and climatized bedrooms were refurnished to provide a comfortable living room atmosphere and equipped with dimmable lights and sound systems. Throughout all study days, a standardized playlist containing non-stimulating background music was played to provide a feeling of comfort and relaxation. An experimenter was always present in the room to supervise the participants.

### 2.3 Study drug

DMT hemifumarate was obtained by basic-organic extraction from the root bark of Mimosa tenuiflora (The Mimosa Company, 1069CL Amsterdam, NL), with n-heptane as the organic solvent. First, shredded root bark was macerated at 50°C in a basic aqueous solution (pH 10; sodium hydroxide) and stirred on the magnetic stirrer for 1 h. Second, heptane was added to the macerate and mixed for 20 min to allow the DMT freebase to transfer into the heptane phase. After that, the heptane was collected. Step one was then repeated with an additional three fresh batches of heptane to maximize DMT yield. Third, the combined heptane fractions were filtered, and the volume was reduced by 50% using a rotary evaporator. Fourth, the concentrated heptane fraction was stepwise cooled down to initiate crystallization (2 h at room temperature, 12 h at 4°C, and 72 h at −20°C). Fifth, the wet DMT freebase crystals were harvested and rinsed with fresh heptane (4°C). Sixth, the crystals were dissolved in a fresh batch of heptane (50°C) and the crystallization process (see step four) was repeated. Seventh, DMT freebase was dissolved in acetone and then an acetonic solution of fumaric acid (hemi equimolar amount of the DMT freebase) was drop-wise added to the DMT solution to form the DMT hemifumarate. The DMT hemifumarate salt was then rinsed with fresh acetone and dried under a vacuum. The final product was subjected to qualitative and quantitative analysis via quantitative Nuclear Magnetic Resonance (qNMR), liquid chromatography-tandem mass spectrometry (LC-MS/MS), and high-performance liquid chromatography (HPLC), revealing a purity of 98.2% ± 0.4%. Harmine hydrochloride (harmine HCl, ≥98% HPLC-tested) was procured from Santa Cruz Biotechnology Inc. (Dallas, Texas 75220, United States).

### 2.4 Peroral DMT formulation

DMT hemifumarate (dose 1: 30 mg; dose 2: 50 mg) was encapsulated into an opaque size 0 hydroxypropyl methylcellulose (HPMC; Interdelta S.A. Givisiez, 1762, Switzerland) capsules, whereas mannitol was used as filler.

### 2.5 Peroral harmine formulation

To prolong the duration of MAO-A inhibition by harmine and thus the half-life of DMT, a harmine formulation with an extended-release (ER) profile was developed. Therefore, 2 types of harmine tablets (5 mm in diameter) were manufactured, either with an immediate-release (IR) profile (no retardation) or an extended-release (ER) profile. Then both tablet types were combined in a capsule to form a combination product, where parts of the drug are released immediately (150 mg), and another proportion (100 mg) is released slowly. Harmine IR minitablets were obtained as follows: Harmine HCl was ground and compressed using a StylOne (Medelpharm, France) tablet press to form IR minitablets containing 25 mg of harmine HCl, 3 mg croscarmellose sodium (AcDiSol, FMC), and 30 mg dicalcium phosphate (Fijicalin, Fuji Chemicals, Japan). Harmine ER minitablets were manufactured as follows: 25 mg harmine HCl was ground, blended with 10 mg hydroxypropylmethylcellulose (Methocel K100, Colorcon, UK) an 27.5 mg Fujicalin, and compressed with a StylOne tablet press, to form ER minitablets containing 25 mg harmine HCl. For study days 1 and 2, six harmine IR tablets (each 25 mg) were combined with four harmine ER tablets (each 25 mg) and encapsulated into an opaque size 0 HPMC capsules.

### 2.6 Dose regimen days 1 and 2

During study days 1 and 2, subjects received both harmine and DMT as peroral formulations. 30 min following peroral premedication with 250 mg harmine HCl (150 mg IR + 100 mg ER), subjects ingested a dose of 30 mg (day 1) or 50 mg (day 2) of DMT hemifumarate.

### 2.7 Intranasal DMT formulation

DMT hemifumarate was aseptically dissolved in NaCl 0.9% to form a nasal spray solution with a 5 mg DMT hemifumarate concentration per puff. The solution was then transferred into nasal spray PUMP systems with a puff volume of 50 μL (Aptar Pharma, 78431 Louveciennes, France). For study day 3, a total of 50 mg (+20% excess) DMT, and for study day 4, a total of 90 mg (+20% excess) was prepared. The 20% excess volume was added to avoid air aspiration and, consequently, dilution of the administered dose.

### 2.8 Harmine buccal formulation

Harmine HCl orodispersible tablets (ODT) for buccal delivery were obtained by freeze-drying. To that, harmine HCl, mannitol, and HPMC were dissolved in deionized water, filled into aluminum blister molds, and freeze-dried for 30 h. For study day 3, two ODTs containing 75 mg harmine HCl each were prepared (total dose of 150 mg). For study day 4, two ODTs containing 100 mg each were manufactured (a total dose of 200 mg).

### 2.9 Dosing regimen days 3 and 4

30 min following buccal premedication with harmine HCl (day 1: 150 mg; day 2: 200 mg), the intranasal repeated-intermittent administration of DMT was initiated. On days 3 and 4, subjects were given the chance to control the psychedelic strength of the experience to enhance safety and tolerability. On day 3, subjects were incrementally given a maximum total dose of 50 mg DMT in intervals of 15 min. On day 4, subjects were incrementally given a maximum dose of 90 mg DMT in intervals of 15 min. At the 1^st^ (0-min), 2^nd^ (15-min) and 3^rd^ (30-min) timepoints, the doses were pre-determined by the experimenter. At the 4^th^ (45-min), 5^th^ (60-min), 6^th^ (75-min), 7^th^ (90-min), 8^th^ (105-min) and 9^th^ (120-min) timepoints, volunteers were allowed to self-determine the dose based on their general wellbeing. A summary of the chosen dose increments is provided in Section 3.1, Table 1.

**TABLE 1:** Summary of the self-determined intranasal DMT dose escalation for study day 3 (top) and 4 (bottom). At the 1st (0-min), 2nd (15-min), and 3rd (30-min) timepoints, the DMT doses were pre-determined by the experimenter (shaded area). At the 4^th^ (45-min), 5^th^ (60-min), 6^th^ (75-min), 7^th^ (90-min), 8^th^ (105-min) and 9^th^ (120-min) timepoints, volunteers were allowed to self-determine the intranasal dose increment based on their general wellbeing. On day 3, either a dose of 0 or 5 mg, and on day 4, either a dose of 0, 5, or 10 mg were allowed. Numbers in the table indicate the number of volunteers that chose the corresponding dose.

Self-determined repeated intermittent dosing regimen									
	Dose increment								
Day 3	1st	2nd	3rd	4th	5th	6th	7th	8th	9th
0 mg	-	-	-	1	1	1	1	0	2
5 mg	-	10	10	9	9	9	9	10	8
10 mg	10	-	-	0	0	0	0	0	0
**Day 4**	1st	2nd	3rd	4th	5th	6th	7th	8th	9th
0 mg	-	-	-	0	0	1	1	1	2
5 mg	-	-	-	1	0	0	1	0	0
10 mg	10	10	10	9	10	9	8	9	8

### 2.10 Blood sampling

Blood samples were collected from the left antecubital vein at −30 (baseline), −15, 0, 15, 30, 45, 60, 90, 120, 180, 240, 300 and 360 min after the first DMT administration for analysis of blood plasma levels of harmine, DMT and the two major DMT metabolites 3-indole acetic acid (3-IAA) and N-methyl-tryptamine (NMT). The venous catheter was connected to a 100 mm Heidelberger plastic tube extension to collect blood samples without disturbing the subjects during their psychedelic experience. The intravenous line was kept patent with a slow drip (10 mL/h) of heparinized saline (1000 IU heparin in 0.9 g NaCl/dL; HEPARIN Bichsel; Bichsel AG, 3,800 Unterseen, Switzerland). Blood samples were immediately centrifuged for 10 min at 2000 RCF. Then, plasma was transferred to Eppendorf tubes, shock-frozen in liquid nitrogen (∼-196°C), and stored at −80°C until assayed.

### 2.11 Analysis of blood levels

DMT was purchased from Lipomed (Arlesheim, Switzerland), NMT and 3-IAA were purchased from Sigma-Aldrich (St. Louis, United States), and harmine, harmol, DMT-N-oxide, harmine-d3 and DMT-d6 were purchased from Toronto Research Chemicals (Toronto, Canada). All other used chemicals were of highest grade available.

For the sample preparation 200 µL of plasma were spiked with 50 µL internal standard (IS) mixture (40 ng/mL DMT-d6 and harmine-d3) and 50 µL methanol (MeOH). Proteins were precipitated by adding 400 µL of acetonitrile (ACN). The samples were shaken for 10 min and centrifuged for 5 min at 10,000 rpm. 350 μL of the supernatant was transferred into an auto-sampler vial, evaporated to dryness under a gentle stream of nitrogen at room temperature and reconstituted in 100 µL eluent-mixture (98:2, v/v). External calibrator and quality control (QC) samples were prepared accordingly, replacing the MeOH with a calibrator or QC solution mixtures. Calibrator and QC samples containing 3-IAA were prepared separately, replacing plasma with water. The calibration ranges were 0.5–500 ng/mL for DMT and DMT-N-oxide, 2.5–120 ng/mL for harmine, 1–80 ng/mL for harmol, 0.015–10 ng/mL for NMT and 35–3,000 ng/mL for 3-IAA.

Samples were analyzed on an ultra-high performance liquid chromatography (UHPLC) system (Thermo Fisher, San Jose, CA) coupled to a linear ion trap quadrupole mass spectrometer 5,500 (Sciex, Darmstadt, Germany). The mobile phases consisted of a mixture of water (eluent A) and ACN (eluent B), both containing 0.1% formic acid (v/v). Using a Kinetex C18 column 50 × 2.1 mm, 2.6 µm (Phenomenex, Aschaffenburg, Germany), the flow rate was set to 0.5 mL/min with the following gradient: starting conditions 98% eluent A, decreasing to 70% within 4 min, followed by a quick decrease to 5% within 1 min, holding for 0.5 min and returning to starting conditions for 1.5 min, resulting in a total runtime of 7 min. The mass spectrometer was operated in positive electrospray ionization mode with scheduled multiple reaction monitoring. The following transitions of precursor ions to product ions were selected as quantifier ions: DMT m/z 189→115, DMT-N-oxide m/z 205→117, harmine m/z 213→169, harmol m/z 199→131, NMT m/z 175→144 and 3-IAA m/z 176→103.

### 2.11 Acute psychometry

Subjective drug effects were assessed throughout the study days at different timepoints using visual analogue scales (VAS, range 0–100) on a touchscreen tablet: Day 1 at 60, 120, 180, and 240 min; day 2 at −15, 0, 15, 30, 45, 60, 90, 120, 180, 240 min; and days 3 and 4 at −15, 0, 15, 30, 45, 60, 75, 90, 105, 120, 135, 180, 240, and 300 min after first DMT administration. Additionally, over all study days, participants were verbally asked about subjective drug effects at 360 min. The VAS included intensity, liking, and arousal, which are presented in this paper; further phenomenological assessments (including additional acute VAS and psychometric instruments) will be presented in a separate publication.

### 2.12 Vital signs and adverse effects

The participants were monitored with regard to adverse effects throughout the experiment by the study physician at baseline, 30, 60, 120, 240, and 480 min after drug administration. Questionnaire-based assessments (VAS, 0–100 or y/n) included physical and mental discomfort, breathing difficulties, racing heartbeat, chest or abdominal pains, unpleasant body sensations/muscle pains, headache, nausea, vomiting, and fainting. Vital signs (systolic/diastolic blood pressure, heart rate, body temperature) were assessed throughout the study at baseline, 30, 90, 150, 210, and 360 min after drug administration for days 1 and 2, and at baseline, 0, 30, 60, 90, 120, 180, 240, and 300 min after drug administration for days 3 and 4.

## 3 Results

### 3.1 Pharmacokinetics

**FIGURE 2:**
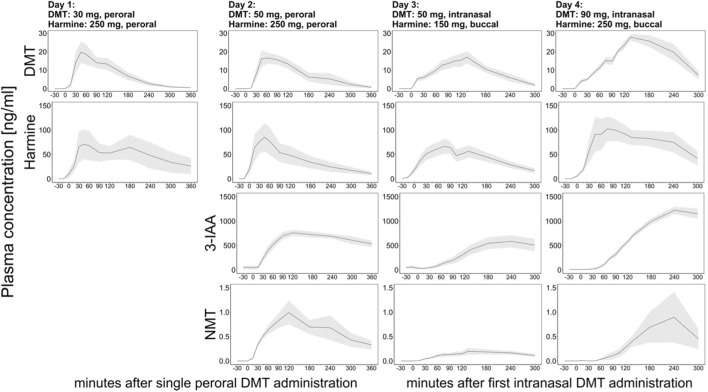
Time course of the mean blood plasma profiles of DMT (1^st^ row), harmine (2^nd^ row), 3-IAA (3^rd^ row), and NMT (4^th^ row) for study day 1 (1^st^ column), 2 (2^nd^ column), 3 (3^rd^ column) and 4 (4^th^ column). Black lines indicate mean analyte concentrations (displayed in ng/mL), and dark shades indicate standard error of the mean (SEM). The *x*-axis displays the time (min) based on the start of DMT administration (timepoint “0”).

The course of the mean plasma concentrations over time of the administered substances DMT and harmine, as well as DMT’s primary metabolites, indole-3-acetic acid (3-IAA), and N-methyltryptamine (NMT), are shown in Figure 2. For day 1, only DMT and harmine were quantified. On days 1 and 2, both DMT and harmine were administered orally (day 1: 30 mg DMT oral, 250 mg harmine oral; day 2: 50 mg DMT oral, 250 mg harmine oral). On days 3 and 4, DMT was administered intranasally via nasal spray, and harmine was delivered buccally via orodispersible tablets (day 3: maximally 50 mg DMT intranasal, 150 mg harmine buccal; day 4: maximally 90 mg DMT intranasal, 200 mg harmine buccal). The intranasal administration schedule was adjustable based on the tolerability of DMT in the individual subjects to increase safety of this first-in-human dose-finding trial. Table 1 displays the number of subjects which were administered each amount of DMT on day 3 and 4. On both days, 8 of 10 subjects were administered the maximum amount of DMT, corresponding to 50 mg on day 3 and 90 mg on day 4.

All 10 study subjects were present and fully participated on each study day. However, on days 1, 2, and 3, plasma samples could only be analyzed from 9, 9, and 8 participants, respectively. On day 4, the plasma samples of all 10 participants were analyzed. Exclusions of the plasma analysis on days 1, 2, and 3 were due to ruptured red blood cells in the plasma, which could have confounded the plasma concentrations of the analyzed substances. None of the subjects had to be excluded because of vomiting, as there were no incidences of vomiting on any of the study days.

As depicted in Figure 2, the peak of the mean concentrations of DMT was slightly higher on day 1 (27.9 ± 15.5 ng/mL) than on day 2 (23.6 ± 11.9 ng/mL), even though more DMT was administered on day 2 via the oral route (day 1: 30 mg DMT, 250 mg harmine; day 2: 50 mg DMT, 250 mg harmine). The peak of the mean concentrations of DMT was similar on days 2 and 3 (23.9 ± 5.0 ng/mL), even though the amount of administered harmine on day 3 was only 60% of the harmine dose on day 2 (day 2: 250 mg oral; day 3: 150 mg buccal) to adjust for higher bioavailability with buccal vs. oral administration. The peak of the mean concentrations of DMT was the highest on day 4 (33.1 ± 9.3 ng/mL).

On day 4, the peak of the mean concentrations of harmine was higher (132.4 ± 111.6 ng/mL) than on day 2 (104.8 ± 100.5 ng/mL), even though on day 4, the amount of administered harmine was only 80% of the harmine dose on day 2 (day 2: 250 mg oral; day 4: 200 mg buccal). On day 3, the peak of the mean concentrations of harmine was lower (93.9 ± 33.5 ng/mL) than on day 1 (125.5 ± 111.2 ng/mL). However, on day 3, the amount of administered harmine was only 60% of the harmine dose of day 1 (day 1: 250 mg oral; day 3: 150 mg buccal). The course of the plasma concentrations of 3-IAA was generally similar on days 2 and 3 and considerably higher on day 4, when the highest amount of DMT was administered, and MAO-enzymes were presumably more strongly inhibited due to higher harmine doses. The concentrations of NMT followed the kinetics of DMT, but were much lower on day 3 than on days 2 and 4, despite similar DMT peak concentration values on days 2 and 3. Even though DMT values were much higher on day 4 than on day 2, the NMT values remained in a similar range on both study days.

### 3.2 Subjective effects

**FIGURE 3:**
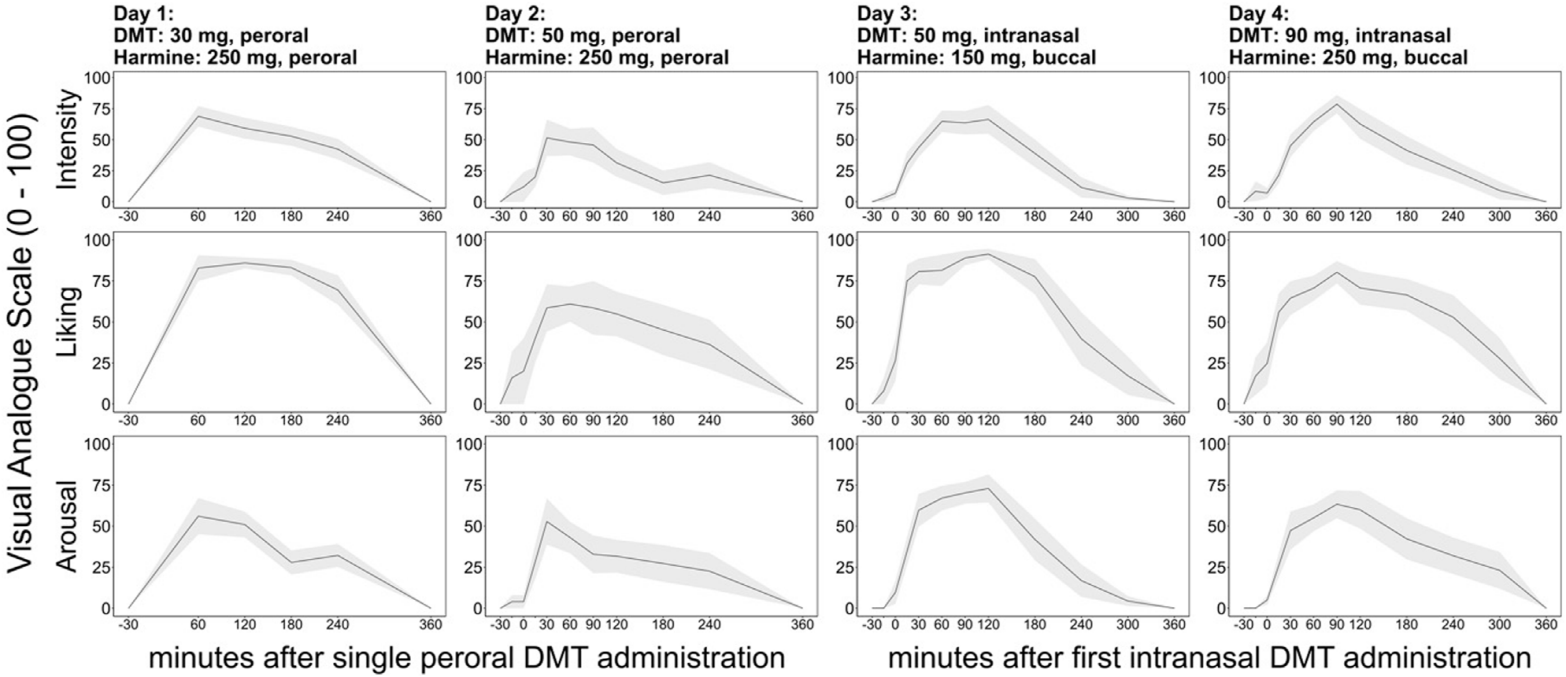
Time course of the VAS intensity (1^st^ row), liking (2^nd^ row), and arousal (3^rd^ row) rating for study day 1 (1^st^ column), 2 (2^nd^ column), 3 (3^rd^ column) and 4 (4^th^ column). Black lines indicate mean ratings on a 100 mm VAS scale (displayed in mm), and dark shades indicate standard error of the mean (SEM). The *x*-axis displays the time (min) based on the start of DMT administration (timepoint “0”).

Subjective effects reported by the participants (VAS-scale) at different timepoints throughout the study days regarding intensity of drug effect, liking of drug effect, and arousal are shown in Figure 3 (D1 and D2: peroral administration of harmine and DMT; D3 and D4: buccal administration of harmine, intranasal administration of DMT). Both forms of administration and all 4 doses induced robust increases in subjective intensity, liking, and arousal. With oral administration, peak effects were reported 60 min after DMT administration on day 1 and 45 min after DMT administration on day 2, followed by a gradual decrease of subjective effects (with a slight plateau effect for liking). Some PD habituation effects were mentioned by participants between sessions 1 and 2 due to increased levels of familiarity with the study procedures and the effect of the strength of the drug, which are reflected in a slight overall decrease in value ratings even though a higher dose of DMT was administered on day 2. Repeated-intermittent intranasal DMT administration (every 15 min until approx. 135 min) on days 3 and 4 led to an extended plateau with maximum scorings of subjective effects between 90–120 min after the first DMT administration. Reported peak effects of intensity reached on average, 69.0% ± 26.3 % of the maximal possible score on day 1, 57.2% ± 39.9 % on day 2, 72.0% ± 21.5 % on day 3, and 79.0% ± 20.8 % on day 4. Reported peak effects of liking reached on average 86.0% ± 10.6 % of the maximal possible score on day 1, 67.1% ± 34.5 % on day 2, 92.0% ± 10.3 % on day 3, 86.0% ± 13.5 % on day 4. Reported peak effects of arousal reached on average 56.1% ± 34.9 % of the maximal possible score on day 1, 55.7% ± 33.1 % on day 2, 73.0% ± 27.1 % on day 3, 63.4% ± 27.0 % on day 4. Acute subjective effects of DMT/harmine gradually subsided 135 min after first DMT administration, but moderate effects lasted up to 300 min. Subjective effects approximately followed the measured blood plasma concentrations of harmine and DMT, particularly on days 3 & 4 with the improved administration routes (buccal harmine, intranasal DMT). More qualitative and phenomenological information on the nature of the effects induced by DMT/harmine will be reported in a different publication.

The coefficient of variation for the individual maximum intensity rating for each day, were as follows: day 1: 37.6; day 2: 51.7; day 3: 28.6; day 4: 23.2

### 3.3 Vital signs

**FIGURE 4:**
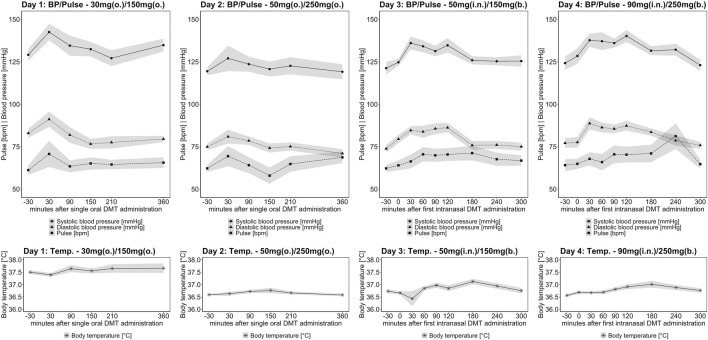
Time course of the vital signs (● systolic BP; ▲ diastolic BP, ◼ pulse, ✷ body temperature) for study day 1 (1^st^ column), 2 (2^nd^ column), 3 (3^rd^ column), and 4 (4^th^ column). Black lines indicate mean values, and dark shades indicate standard error of the mean (SEM). The *x*-axis displays the time (min), based on the start of DMT administration (timepoint “0”).

Mean values for body temperature (BT), systolic blood pressure (SBP), diastolic blood pressure (DBP), and heart rate over time are shown in Figure 4. In general, the administration of DMT and harmine induced low to moderate elevations of the cardiovascular parameters. Mean heart rate values stayed in the range of 60–80 bpm. SBP increased on average by 15 mmHg. The peak values were observed 30 min (days 1, 2, 3) and 120 min after DMT administration (day 4). DBP changed by 10 mmHg with peak levels at 30 min after DMT administration. The maximal change of BT was 1°C, with no levels above 37.8°C. The higher baseline BT values on day 1 might be related to adjustments of systemic room temperature in the laboratories and the exchange of BT measuring devices after the first session, which may have caused some measuring artifacts.

### 3.4 Undesired drug effects

In Table 2, the frequency and mean intensity of undesired drug effects are shown for all study days. Assessed were somatic distress, psychological distress, difficulty breathing, heart racing, chest pain, stomachache, muscle aches, headache, nausea, and fainting at timepoints 60, 120, 180, and 240 min after DMT administration. Subjects could choose a value from 0 “no effect” to 100 “strong” effect on a VAS scale. Most undesired drug effects (i.e., low to moderate levels of nausea and somatic discomfort) were observed in the first two study days with oral DMT/harmine administration, particularly during the first 2 hours after dosing. Repeated intermittent doses of intranasal DMT combined with buccal harmine were better tolerated, with fewer reported adverse effects on study days 3 and 4. All adverse effects were transient and resolved within the sampling intervals and no additional medical intervention was needed to ensure participant’s safety.

**TABLE 2:** Frequency (i.e., number of participants reporting undesired drug effects), mean intensity values (visual analogue scales), and standard deviations (displayed in brackets) are reported for study day 1 (250 mg oral harmine; 30 mg single oral DMT dose; top left), study day 2 (250 mg oral harmine; 50 mg single oral DMT; bottom left), study day 3 (150 mg oromucosal harmine; 50 mg repeated-intermittent intranasal DMT; top right) and study day 4 (200 mg oromucosal harmine; 90 mg repeated-intermittent intranasal DMT; bottom right). Values greater than zero are highlighted in grey (ranged 0%–100%).

	minutes after single oral DMT intake					minutes after first intranasal DMT administration			
	60	120	180	240		60	120	180	240
Day 1					Day 3				
Somatic distress	3; 33 (26)	2; 7 (7)	0; 0 (0)	0; 0 (0)	Somatic distress	2; 10 (9)	0; 0 (0)	0; 0 (0)	0; 0 (0)
Psychological distress	1; 70 (0)	0; 0 (0)	0; 0 (0)	0; 0 (0)	Psychological distress	2; 15 ± (13)	0; 0 (0)	0; 0 (0)	0; 0 (0)
Breathing difficulty	2; 7 (4)	0; 0 (0)	0; 0 (0)	1; 4 (0)	Breathing difficulty	0; 0 (0)	0; 0 (0)	0; 0 (0)	0; 0 (0)
Heart racing	1; 52 (0)	1; 15 (0)	1; 10 (0)	1; 6 (0)	Heart racing	1; 4 (0)	0; 0 (0)	0; 0 (0)	0; 0 (0)
Chest pain	1; 16 (0)	1; 17 (0)	0; 0 (0)	0; 0 (0)	Chest pain	1; 66 (0)	0; 0 (0)	2; 55 (13)	0; 0 (0)
Stomach ache	1; 39 (0)	0; 0 (0)	0; 0 (0)	0; 0 (0)	Stomach ache	1; 2 (0)	0; 0 (0)	0; 0 (0)	0; 0 (0)
Muscle ache	3; 30 (17)	0; 0 (0)	0; 0 (0)	0; 0 (0)	Muscle ache	0; 0 (0)	0; 0 (0)	0; 0 (0)	0; 0 (0)
Head ache	1; 28 (0)	0; 0 (0)	0; 0 (0)	0; 0 (0)	Head ache	0; 0 (0)	0; 0 (0)	1; 28 (0)	1; 7 (0)
Nausea	3; 22 (13)	2; 54 (66)	0; 0 (0)	0; 0 (0)	Nausea	0; 0 (0)	0; 0 (0)	0; 0 (0)	0; 0 (0)
Fainting	1; 17 (0)	1; 64 (0)	1; 78 (0)	0; 0 (0)	Fainting	0; 0 (0)	1; 48 (0)	1; 53 (0)	0; 0 (0)
Day 2					Day 4				
Somatic distress	3; 49 (37)	1; 20 (0)	0; 0 (0)	0; 0 (0)	Somatic distress	1; 12 (0)	3; 31 (38)	0; 0 (0)	1; 11 (0)
Psychological distress	2; 51 (16)	2; 6 (4)	0; 0 (0)	0; 0 (0)	Psychological distress	0; 0 (0)	0; 0 (0)	0; 0 (0)	1; 22 (0)
Breathing difficulty	0; 0 (0)	0; 0 (0)	0; 0 (0)	0; 0 (0)	Breathing difficulty	0; 0 (0)	0; 0 (0)	0; 0 (0)	0; 0 (0)
Heart racing	1; 82 (0)	0; 0 (0)	1; 61 (0)	0; 0 (0)	Heart racing	0; 0 (0)	0; 0 (0)	1; 44 (0)	0; 0 (0)
Chest pain	1; 4 (0)	0; 0 (0)	0; 0 (0)	0; 0 (0)	Chest pain	0; 0 (0)	0; 0 (0)	0; 0 (0)	0; 0 (0)
Stomach ache	2; 25 (33)	0; 0 (0)	0; 0 (0)	0; 0 (0)	Stomach ache	0; 0 (0)	0; 0 (0)	0; 0 (0)	0; 0 (0)
Muscle ache	0; 0 (0)	0; 0 (0)	0; 0 (0)	0; 0 (0)	Muscle ache	0; 0 (0)	1; 12 (0)	0; 0 (0)	0; 0 (0)
Head ache	0; 0 (0)	0; 0 (0)	0; 0 (0)	0; 0 (0)	Head ache	1; 23 (0)	0; 0 (0)	0; 0 (0)	1; 25 (0)
Nausea	3; 59 (37)	2; 16 (8)	0; 0 (0)	0; 0 (0)	Nausea	1; 25 (0)	2; 52 (40)	1; 13 (0)	0; 0 (0)
Fainting	1; 34 (0)	1; 57 (0)	0; 0 (0)	0; 0 (0)	Fainting	0; 0 (0)	0; 0 (0)	0; 0 (0)	0; 0 (0)

In Table 3, the individual liver enzymes (ASAT/GOT, ALAT/GPT and GAMMA-GT) values are shown at baseline (−30 min) and after the session (300 min) for study day 4, when the largest dose was administered. No clinically relevant change in liver enzyme levels was noted in any subject before or after the dosing session.

**TABLE 3:** Subject’s individual liver enzyme values (in units per L; U/L) at baseline (−30 min) and after the session (300 min) are displayed for day 4 (largest dose). ASAT/GOT, aspartate aminotransferase/glutamic oxaloacetic transaminase; ALAT/GPT, alanine transaminase/glutamate-pyruvate transaminase; GAMMA-GT, gamma-glutamyl transferase.

Liver enzymes										
Day 4	Subject (#)									
Timepoint	Analyt	Reference	#3	#4	#5	#6	#7	#8	#9	#10
Baseline (t_-30_)	ASAT/GOT (U/L)	**<37**	17	22	21	17	23	17	17	20
	ALAT/GPT (U/L)	**<65**	18	16	23	24	20	24	16	31
	GAMMA-GT (U/L)	**<85**	17	15	17	18	16	18	14	14
After session (t_300_)	ASAT/GOT (U/L)	**<37**	19	25	20	29	23	18	16	20
	ALAT/GPT (U/L)	**<65**	17	17	23	25	20	25	16	36
	GAMMA-GT (U/L)	**<85**	16	15	16	18	17	19	12	16

The bold numbers indicate the clinical threshold values. Concentrations higher than the reference values indicate a clinically relevant increase in the liver enzyme concentration.

## 4 Discussion

In this first-in-human pilot study, we tested PKPD characteristics of different innovative DMT/harmine formulations specifically designed to overcome clinical limitations associated with the use of traditional botanical ayahuasca. We developed and tested an oral vs. parenteral formulation of DMT and harmine at two dose levels each in an open-label within-subject dose-finding study in 10 healthy male subjects through continuous blood sampling, vital sign monitoring, and psychometric assessments.

Both oral DMT/harmine doses (day 1: 250 mg harmine +30 mg DMT; day 2: 250 mg harmine +50 mg DMT) were tolerated well by all study volunteers. Up to 30% of participants experienced transient and dose-dependent adverse effects of low to moderate intensity (e.g., nausea and somatic discomfort) during the first 2 hours after dosing, which resolved spontaneously within the assessment interval. Similar to other serotonergic psychedelics such as psilocybin or LSD (Passie et al., 2002; Passie et al., 2008), a mild and asymptomatic increase in blood pressure was observed for both oral doses, which may be triggered by peripheral stimulation of the sympathetic nervous system via the modulation of noradrenergic and 5-HT_2A_ receptors (Watts et al., 2012). While vomiting and diarrhea are common features of traditional botanical ayahuasca, no such cases were observed in our study sample. The attenuation of GI side effects is likely to result from the lower cumulative alkaloid load in the standardized DMT/harmine formula compared to herbal ayahuasca preparations—which contain high amounts of other serotonergic compounds, such as harmaline and tetrahydroharmine. Thus, well-balanced doses of purified DMT and harmine may spare 5-HT_3_ chemoreceptors in the GI tract and thus yield less nausea and vomiting. Moreover, the absence of emetogenic plant tannins in purified DMT/harmine formulations may also favor GI tolerability.

Surprisingly, we found huge interindividual PK differences DMT and harmine plasma concentrations after a single oral administration. Thereby, peak plasma concentrations (C_max_) varied by a factor of ∼6.8 for DMT and even a factor of ∼55.5 for harmine across subjects, and likewise, time to peak values (t_max_) of harmine ranged from 60 to 270 min. There was no clear PK-related dose-response effect for DMT with oral dosing, which underscores that this route of administration is rather unpredictable and associated with high inter- and intraindividual variability. Consistently, subjective drug effects varied dramatically between subjects, for example, on day 1 with peak intensity ratings ranging from 6 (non-responder; n = 1), (intermediate responders’ intensity between 30 and 80; n = 4) to 100 (full responders’ intensity >80; n = 5), whereby some full responders reported after the session that maximum intensity ratings during the DMT peak were quite overwhelming (qualitative interview data will be reported in a separate publication).

Even though oral DMT/harmine was associated with fewer side effects than typically reported after traditional botanical ayahuasca consumption (Bouso et al., 2022), the considerable differences in interindividual PK profiles and subjective responsiveness raised doubts, whether oral DMT/harmine preparations would ever meet the regulatory requirements regarding risk/benefit and safety/tolerability assessment for clinical use in patient populations. Based on our PK results, we suggest two mechanisms to account for the observed findings: First, in case of insufficient inhibition of the GI MAO-A enzyme, DMT is immediately degraded following oral ingestion. Since the density of endothelially expressed MAO-A enzymes (Anderson et al., 1993)—and consequently the magnitude of this “endothelial first-pass effect”—can vary across subjects, the harmine dose required to yield sufficient MAO-A inhibition may as well vary across individuals. Moreover, harmine is degraded by the hepatic enzyme CYP2D6 and other CYP450 isoenzymes (Yu et al., 2003). Multiple allelic variants of the CYP2D6 gene have been identified, which are associated with reduced or increased enzyme activity in individuals who are respectively so-called poor metabolizers (PMs), extensive metabolizers (EMs), and ultrarapid metabolizers (UMs) (Peñas-Lledó and Llerena, 2014). Thus, depending on an individual’s allelic variant of the CYP2D6 gene, the bioavailability of harmine may vary substantially across subjects. Notably, in previous studies with herbal ayahuasca, rapid vs. slow metabolizers were distinguished based on interindividual differences in plasma harmine levels (Callaway, 2005).

Parenteral administration of DMT (e.g., intravenous) results in short-acting psychotropic effects, whereas even high doses of orally administered DMT do not produce any somatic or psychological effects. This observation implies a robust first-pass metabolism in the intestine, likely due to the dense expression of MAO-A enzymes on the apical cells of the GI tract. Remarkably, studies in rats have shown that shortly after intraperitoneal injection, DMT accumulates in the brain, achieving a brain-to-blood partition ratio of approximately 5:1 (Cohen and Vogel, 1972; Sitaram et al., 1987). A recent study in rats further suggested a potential involvement of neuronal MAO-A enzymes in the metabolism of DMT into 3-IAA. Consistently, co-administration of harmine and DMT resulted in a 50% reduction in cerebral 3-IAA levels, which may indicate a MAO-A inhibition within the living rat brain (Egger et al., 2023). Anyhow, whether cerebral 3-IAA levels reflect central or peripheral breakdown of DMT remains unknown. Given the high density of MAO-A enzymes in the intestine, oral ingestion of DMT and harmine may necessitate high luminal GI concentrations of harmine to enable the oral bioavailability of DMT, whereas lower levels of harmine may suffice to prevent DMT’s deamination by mitochondrial MAO-A, once DMT has entered the bloodstream. Therefore, when DMT is administered parenterally, lower doses of harmine may be sufficient to extend the half-life of DMT compared to oral formulations.

Thus, circumventing the GI tract may offer a dose-sparing option, that overcomes both PK (high intersubject variability) and PD (GI discomfort) shortcomings of oral DMT/harmine and thus increase overall predictability and tolerability. Based on this notion, we developed and tested an innovative combination kit comprising a DMT nasal spray that enables the parenteral, metered application of small dose increments (5 mg/puff) and a harmine-containing ODT for buccal delivery over the oromucosal route. Unlike the oral formula, the parenteral combination yielded more homogenous PK profiles, whereby peak plasma concentrations (c_max_) varied by a factor of ∼2.3 for DMT and only a factor of ∼3.2 for harmine across subjects. Consistently, response predictability was much higher compared to the oral preparations, such that all volunteers responded to the parenteral delivery with intensity plateaus in the range of 7–10 out of 10. Interestingly, the buccal delivery of harmine produced an even smoother sustained-release profile compared to the combined IR/ER oral harmine formulation, which is favorable for extended repeated intermittent dosing of DMT over 120 min.

Most importantly, the self-determined, repeated-intermittent administration of DMT via nasal spray—as implemented for this dose-finding pilot trial—in principle allows adjusting the dosing regimen according to individual tolerability, rendering this approach much more controllable and thus safer to guide the participants through the psychedelic experience. Due to improved bioavailability with parenteral dosing, subjective effects are more predictable with less variance between participants and more reliable PKPD time courses. The parenteral combination kit was tolerated extremely well, with considerable attenuation of adverse effects compared to the oral DMT/harmine formula or reported adverse effects after traditional herbal ayahuasca consumption, which may range up to 69.9% according to a large representative survey (n = 10,836) (Bouso et al., 2022). Likewise, we observed a mild and asymptomatic increase in blood pressure. This participant-controlled administration of a psychedelic compound is new and appears particularly valuable for future applications in psychiatric patients, given the need to minimize the psychological risks inherent with the use of psychedelics in mental healthcare. Anyhow, a prerequisite for this flexible dosing approach is a short half-life of the psychedelic compound needed to dynamically control the subjective effect profile—and is thus withheld to short-acting compounds like DMT.

In sum, the combined parenteral administration of DMT and harmine is an innovative approach that may pave the way towards a safe, rapid-acting, and patient-oriented treatment of affective disorders.

## 5 Limitations

When interpreting the results of this study, it is important to consider several limitations inherent to the experimental design and methodology. First, the present study followed an open-label dose escalation design in a small sample of male subjects, whereby subjects were informed on each study day about the intervention they would receive. Thus, the presence of significant expectation effects cannot be ruled out. Likewise, since the order of the interventions was not randomized, order and PD habituation effects—such as an increased excitement on the first study day—are probable. In this study, we combined intranasal DMT with various doses of harmine. However, we did not investigate the effects of intranasal administrations of DMT without harmine. Consequently, it remains unclear what pharmacological effects intranasal DMT might elicit on its own. In the 1950s, Turner and Merlis conducted a study which found that intranasal administration of DMT freebase in the dose range of 5–20 mg was ineffective (Turner and Merlis, 1959). In our study, we administered total doses of 50 mg (Day 3) and 90 mg (Day 4) of DMT as a hemifumarate salt, equivalent to approximately 38 mg (Day 3) and 70 mg (Day 4) in freebase form. Additionally, DMT hemifumarate is likely to be absorbed more efficiently compared to the freebase due to its superior aqueous solubility. Consequently, it is possible that intranasal administration of DMT hemifumarate could produce effects even in the absence of harmine. However, it is important to note that in our study, the total DMT dosage was not administered as a single bolus but in a repeated-intermittent manner over a 2-h time span with dose increments of 5 mg (Day 3; approximately 3.8 mg freebase) and 10 mg (Day 4; approximately 7.7 mg freebase). Thus, given the small dose increments, the 15-min intervals between administrations, and the very short plasma elimination half-life of DMT, approximately 5–10 min (Vogt et al., 2023), it seems rather unlikely that this dosing regimen would have produced substantial DMT effects in the absence of MAO inhibition.

Lastly, the analysis of the study results is reported in a purely descriptive manner and was meant to explore different pharmaceutical approaches to administer DMT/harmine in a reliable and safe manner. Thus, future studies following a placebo-controlled, double-blinded, randomized, crossover design, to allow for statistical analysis are urgently needed.

## Data Availability

The datasets presented in this article are not readily available because Licensing agreement between Reconnect Labs and University of Zurich. Requests to access the datasets should be directed to DD, dario-do@hotmail.com.
